# Association Between Daily Sitting Time and Cardiometabolic Indicators in Adult Public Servants: A Cross‐Sectional Study With Crude and Adjusted Analyses

**DOI:** 10.1002/hsr2.73256

**Published:** 2026-09-14

**Authors:** Leonardo Lima, Carlos Eduardo Rosa da Silva, Erinaldo Andrade, Luis Carlos de Oliveira, Willian Santana, Jose Eduardo Hydes, Yuri Xavier, Aylton Figueira

**Affiliations:** ^1^ GETAFIS—São Judas Tadeu University São Paulo Brazil

## Abstract

**Objectives:**

To investigate the association between daily sitting time (ST) and cardiometabolic indicators in adult Brazilian public servants, using both unadjusted and covariate‐adjusted analyses.

**Methods:**

Cross‐sectional study conducted with 60 adult federal public servants (34 women, 26 men; mean age 43.40 ± 11.42 years) from Caraguatatuba, São Paulo, Brazil. ST was assessed using the short‐form International Physical Activity Questionnaire, and participants were categorized as high ST (≥ 8 h/day, *n* = 21) or low ST (< 8 h/day, *n* = 39). Cardiometabolic indicators included body mass index (BMI), waist‐to‐hip ratio (WHR), systolic (SBP) and diastolic blood pressure (DBP), resting heart rate, peripheral oxygen saturation (SpO_2_), and fasting capillary glucose. Unadjusted associations were examined using Spearman correlations with Fisher z‐transform 95% confidence intervals and Mann–Whitney comparisons between groups. Adjusted analyses included multiple linear regression (MLR) and partial Spearman correlations by the residual method, controlling for sex, age, physical activity, smoking, alcohol use, medication use, pre‐existing conditions, and perceived stress. Prevalence ratios (PR) were estimated using clinically validated cutoff points. The study was approved by the Research Ethics Committee of Universidade São Judas Tadeu (protocol no. 6.117.542).

**Results:**

The high ST group presented significantly higher values of BMI (29.26 ± 4.46 vs. 26.38 ± 4.09 kg/m^2^; *p* = 0.020), WHR (0.86 ± 0.10 vs. 0.80 ± 0.07; *p* = 0.011), SBP (138.33 ± 10.66 vs. 128.23 ± 9.59 mmHg; *p* = 0.003), DBP (89.29 ± 5.87 vs. 85.38 ± 4.07 mmHg; *p* = 0.016), and fasting glucose (101.14 ± 5.67 vs. 96.67 ± 3.37 mg/dL; *p* = 0.001), as well as lower SpO_2_ (93.81 ± 3.59 vs. 96.18 ± 2.30%; *p* = 0.008). Spearman correlations identified significant associations between ST and WHR (*ρ* = 0.278; 95% CI: 0.026–0.497; *p* = 0.031), SBP (*ρ* = 0.290; 95% CI: 0.039–0.507; *p* = 0.024), SpO_2_ (*ρ* = −0.363; 95% CI: −0.565 to −0.121; *p* = 0.004), and fasting glucose (*ρ* = 0.351; 95% CI: 0.107–0.556; *p* = 0.006). Fasting glucose was the only indicator that remained associated with ST after full covariate adjustment (partial *ρ* = 0.279; 95% CI: 0.025–0.499; *p* = 0.032), though the association was not confirmed in multiple regression (*B* = 1.905; 95% CI: −0.256 to 4.066; *p* = 0.083). Prevalence ratio analyses showed that the high ST group had significantly greater prevalence of elevated WHR by sex‐specific WHO criteria (PR = 6.19; 95% CI: 1.91–20.07; *p* = 0.001), low SpO_2_ below 95% (PR = 2.79; 95% CI: 1.36–5.73; *p* = 0.010), and impaired fasting glucose by ADA 2024 criteria (PR = 3.45; 95% CI: 1.63–7.30; *p* = 0.002).

**Conclusions:**

Higher daily sitting time was associated with a less favorable cardiometabolic profile in adult public servants, with significant differences across multiple indicators in unadjusted analyses. Fasting glucose showed the most consistent signal after covariate adjustment, suggesting a possible adjusted association between sitting time and glycemic metabolism, though not confirmed in multiple regression. These findings should be interpreted as exploratory given the cross‐sectional design, restricted sitting time variability, and moderate sample size. Longitudinal studies with objective sedentary behavior measures are needed to confirm the independence and magnitude of these associations.

## 1 Introduction

Sedentary behavior is defined as any activity in a waking state characterized by low energy expenditure (≤ 1.5 METs) in the sitting, reclining, or lying posture [1]. Total daily sitting time is one of the most frequently measured components of sedentary behavior in epidemiological studies and has been consistently associated with increased risk of all‐cause mortality, cardiovascular disease, type 2 diabetes, and hospitalizations in adults, regardless of physical activity level [2, 3].

Population analyses from 54 countries estimate that sitting time greater than 3 h per day is responsible for 3.8% of all‐cause deaths, accounting for approximately 433,000 preventable deaths annually [4]. Cardiovascular diseases are the leading causes of death in the world [5], and in Brazil they account for the highest death rates among chronic non‐communicable diseases, with an estimated economic impact of 266.9 billion dollars annually. National data reveal a high prevalence of sedentary behavior among Brazilian adults, especially in individuals with higher education and income, a profile compatible with that of the public servants evaluated in the present study [6].

From a physiological point of view, prolonged sitting suppresses the contractile activity of the postural muscles and lower extremities, reducing muscle lipoprotein lipase (LPL) activity and GLUT‐4 translocation, central mechanisms in lipid and glycemic metabolism [7]. Pinto et al. [8] demonstrated that these effects configure a physiology specific to sedentary behavior, distinct from the effects of physical inactivity, with consequences on endothelial function, peripheral vascular resistance, and glycemic control. Bergouignan et al. [9] experimentally confirmed that only 3 days of regular interruptions of sitting time with light activity were sufficient to modulate insulin‐independent glucose uptake pathways in skeletal muscle.

Prospective studies and meta‐analyses provide robust evidence on the role of ST in elevating blood pressure and the risk of glycemic changes. Beunza et al. [10] followed 6742 healthy university students and demonstrated that the most sedentary students had a 48% higher risk of developing arterial hypertension, regardless of the level of physical activity. A scoping review of 40 systematic reviews [11] confirmed a positive association between sedentary time and hypertension in cross‐sectional observational studies. In the field of glucose metabolism, a meta‐analysis by Wilmot et al. [3] demonstrated that each additional hour of daily sedentary time increases the risk of type 2 diabetes by 22%, while a systematic review by Cavallo et al. [12] identified that reallocation of ST to light activity consistently improves blood glucose and insulin sensitivity.

A scoping review published in Applied Physiology, Nutrition, and Metabolism [13] concluded that sedentary behavior is associated with worse cardiometabolic outcomes, higher risk of metabolic syndrome, and poorer glycemic control. Additionally, a dose‐response meta‐analysis published in Obesity Reviews [14] demonstrated that prolonged sedentary behavior is associated with a 71% higher risk of metabolic syndrome in the categories of highest exposure, regardless of physical activity.

Despite this growing body of international evidence, Brazilian studies that investigate sitting time and multiple cardiometabolic indicators in working adults in an integrated manner, controlling for confounding variables such as smoking, medication use, and preexisting diseases, are still scarce [15, 16]. The present study aimed to investigate the relationship between daily sitting time and cardiometabolic indicators in adult public servants, in crude and adjusted analyses for clinical and behavioral covariates. It was hypothesized that more daily sitting time would be associated with worse cardiometabolic indicators, especially blood pressure, central adiposity, and glycemia, in the crude analyses.

## 2 Methods

This is an epidemiological, descriptive, cross‐sectional study conducted between March and May 2024 with 60 adult civil servants (34 women and 26 men) of the Federal Justice in Caraguatatuba, São Paulo State, Brazil. The project was approved by the Research Ethics Committee of the São Judas Tadeu University (opinion No. 6,117,542), in accordance with Resolution No. 466/12 of the National Health Council and the principles of the Declaration of Helsinki. Of 72 eligible employees identified at the time of data collection, 60 agreed to participate (response rate: 83.3%). All participants worked under a standardized 8‐h administrative workday as per Brazilian federal civil service regulations (Lei n° 8.112/1990). Individuals with a diagnosis of established cardiovascular diseases, diabetes mellitus, and pregnant women were excluded. Regular medication use was recorded as a covariate, without exclusion by pharmacological class, given that most participants used medications for non‐cardiovascular conditions.

### 2.1 Assessment of Sitting Time and Covariates

Sitting time was evaluated using the IPAQ short version, validated for the Brazilian population [17]. The instrument assesses the total time spent sitting on a typical weekday, with responses expressed in hours per day. In the sample studied, sitting time presented only three distinct values (6, 7.5, and 8 h/day), which reflects the nature of the instrument's responses and resulted in low variability in exposure—a limitation discussed below. For categorical analyses, the threshold of 8 h/day was adopted, frequently used in epidemiological studies and compatible with evidence indicating increased risk in high volumes of sedentary behavior [18, 19]. The covariates collected by a structured questionnaire were: gender, age, level of physical activity (min/wk), smoking (yes/no), alcohol consumption (yes/no), regular use of medications (yes/no), self‐reported preexisting diseases (yes/no), and self‐reported stress level (low/medium/high, ordinally coded for the analysis).

### 2.2 Cardiometabolic Measures

BMI calculated as weight (kg)/height^2^ (m^2^). WHR obtained by dividing waist circumference (measured at the midpoint between the last rib and iliac crest) by hip circumference (at the widest gluteal protrusion), using a non‐elastic metal tape measure. WHR was chosen over waist‐to‐height ratio based on its established use in the Brazilian occupational health literature and the availability of sex‐specific cardiovascular risk cutoff points validated by the WHO [20]. Resting blood pressure and HR were measured with a Microlife BP 3AC1‐1, at rest while sitting, with three consecutive measurements and the average of the last two. Peripheral oxygen saturation by pulse oximetry (SB100 MD), used as an exploratory physiological marker, since SpO_2_ can be influenced by smoking, respiratory conditions, peripheral perfusion, and technical measurement factors. Capillary glucose after fasting for at least 8 h (Accu‐Chek point‐of‐care glucometer (Roche) performs a single fingerstick blood glucose measurement. It is not a continuous glucose monitoring device. Fasting of at least 8 h was verified by self‐report at collection. Used as a field glycemic screening marker, not a definitive clinical diagnosis.

### 2.3 Statistical Analysis

The analyses were performed in SPSS v.26.0 (IBM Corp., USA). Normality was assessed by the Shapiro–Wilk test. Comparisons between groups (ST ≥ 8 h/day vs. ST < 8 h/day) were performed using the bilateral Mann–Whitney test. Categorical variables between the sexes were compared using Pearson's *χ* ^2^ test or Fisher's exact test when the expected frequency in any cell was less than 5. Crude associations were investigated by Spearman's coefficient with 95%CI estimated by Fisher's transformation z, a method consistent with Spearman's *p* (CI excludes zero when *p* < 0.05). For the adjusted analyses: (a) multiple linear regression with ST as the independent variable and covariates sex, age, PA, smoking, alcohol, medications, preexisting diseases, and stress; (b) Spearman's partial correlations by the residue method, with 95%CI by the Fisher z transformation applied to the residuals. Multicollinearity was assessed by the VIF (threshold < 10). The prevalence ratio (PR) was calculated as a simple ratio between prevalences, with 95%CI using the log‐normal method and *p* using Pearson's *χ* ^2^ test or Fisher's exact test when any cell had an expected frequency lower than 5. Poisson regression with robust variance was not used due to the small number of events in some categories. Given the exploratory nature of the study and the number of comparisons performed with multiple outcomes, the results should be interpreted with caution, prioritizing consistency between magnitude of effect, direction of associations, and biological plausibility. Nominal significance: 5%.

## 3 Results

Participants were 60 adults (34 women, 26 men), with a mean age of 43.40 ± 11.42 years and mean sitting time of 7.40 ± 0.71 h/day (range: 6–8 h/day). The entire sample had ST of at least 6 h/day, and 21 participants (35.0%) had ST ≥ 8 h/day. Table 1A presents the general characteristics stratified by sex, and Table 1B presents characteristics stratified by ST group (≥ 8 h/day vs. < 8 h/day). Men had higher ST (7.63 ± 0.54 vs. 7.22 ± 0.77 h/day; *p* = 0.016), higher BMI (28.62 ± 3.37 vs. 26.45 ± 4.90 kg/m^2^; *p*= 0.046) and higher WHR. No significant differences were observed between the sexes for the categorical variables evaluated. Table 1B presents sample characteristics stratified by ST group. The high ST group had significantly greater prevalence of smokers (61.9% vs. 30.8%; *p* = 0.040), medication use (61.9% vs. 25.6%; *p* = 0.013), and poorer sleep quality (52.4% good sleep vs. 87.2%; *p* = 0.008). Mean sleep duration did not differ significantly between groups (7.5 h/night in both groups; *p* = 0.642).

**Table 1A:** General and anthropometric characteristics, sitting time, and covariate profile of adult employees of the Federal Court of Caraguatatuba—São Paulo (mean ± SD or %).

Variable	General (*n* = 60)	Women (*n* = 34)	Men (*n* = 26)	*D*%	Diff. average	95%CI	*p* value
Age (years)	43.40 ± 11.42	40.88 ± 11.89	46.69 ± 10.07	14.2	5.81	(0.13; 11.49)	0.046
Height (m)	1.68 ± 0.07	1.66 ± 0.06	1.71 ± 0.06	2.9	0.05	(0.01; 0.08)	0.006*
Weight (kg)	77.80 ± 14.38	73.21 ± 14.97	83.81 ± 11.23	14.5	10.60	(3.83; 17.37)	0.004*
BMI (kg/m^2^)	27.39 ± 4.41	26.45 ± 4.90	28.62 ± 3.37	8.2	+2.18	(0.04; 4.32)	0.046*
WHR	0.82 ± 0.09	0.78 ± 0.08	0.87 ± 0.07	10.8	0.08	(0.05; 0.12)	< 0.001*
PA (min/wk)	119.67 ± 59.50	127.65 ± 53.83	109.23 ± 65.81	14.4	–18.42	(–50.18; 13.35)	0.238
TS (h/day)	7.40 ± 0.71	7.22 ± 0.77	7.63 ± 0.54	5.7	0.41	(0.08; 0.75)	0.016*
Categorical variables (%/*n*)							

Variable	Overall %	*n*	Women %	*n*	Men %	*n*	*p* value
Smoker (yes)	40.0%	24	42.4%	14	38.5%	10	0.758
Alcoholic beverage (yes)	73.3%	44	64.7%	22	84.6%	22	0.140
Medications (yes)	38.3%	23	32.4%	11	46.2%	12	0.276
Preex diseases (yes)	31.7%	19	20.6%	7	46.2%	12	0.054
Osteoarticular pain	30.0%	18	35.3%	12	23.1%	6	0.306
Previous COVID‐19	33.3%	20	35.3%	12	30.8%	8	0.729
Medium/high stress	68.3%	41	73.5%	25	61.5%	16	0.316

Abbreviations: Δ% = percentage difference between the means of the sexes, BMI = body mass index, PA = physical activity, WHR = waist‐to‐hip ratio, ST = sitting time.

* *p *< 0.05 for the comparison between genders (Student's *t*‐test or Mann–Whitney test for continuous variables; Pearson's *χ* ^2^ test for categorical variables). For the variable Alcoholic beverages, Fisher's exact test was used as an expected frequency ratio of less than 5 in any cell (*p* = 0.140). For prior COVID‐19, *n* = 58 (two participants did not respond to the item; *p* = 0.729).

**Table 1B:** Sample characteristics stratified by daily sitting time group (ST ≥ 8 h/day vs. ST < 8 h/day).

Variable	ST ≥ 8 h/day (*n* = 21)	ST < 8 h/day (*n* = 39)	*p* value
Age (years)	50.0 (44.0–56.0)	39.0 (35.0–49.0)	0.050
Height (m)	171.48 ± 7.04	166.72 ± 6.10	0.013*
Weight (kg)	85.95 ± 13.09	73.41 ± 13.21	0.001*
BMI (kg/m^2^)	29.26 ± 4.46	26.38 ± 4.09	0.019*
WHR	0.86 ± 0.10	0.80 ± 0.07	0.015*
Sleep (h/night)	7.5 (5.0–9.0)	7.5 (6.2–7.5)	0.642
PA (min/wk)	0.0 (0.0–150.0)	150.0 (112.5–150.0)	< 0.001*
ST (h/day)	8.0 (8.0–8.0)	7.5 (6.0–7.5)	< 0.001*
Sleep quality (good) (%)	52.4% (*n* = 11)	87.2% (*n* = 34)	0.008*
Smoker (%)	61.9% (*n* = 13)	30.8% (*n* = 12)	0.040*
Alcohol (%)	76.2% (n = 16)	71.8% (*n* = 28)	0.951
Medications (%)	61.9% (*n* = 13)	25.6% (*n* = 10)	0.013*
Preexisting diseases (%)	47.6% (*n* = 10)	23.1% (*n* = 9)	0.097
Osteoarticular pain (%)	47.6% (*n* = 10)	20.5% (*n* = 8)	0.059
Med/high stress (%)	66.7% (*n* = 14)	69.2% (*n* = 27)	1.000

Abbreviations: BMI = body mass index, PA = physical activity, ST = sitting time, WHR = waist‐to‐hip ratio.

* *p *< 0.05. Continuous variables: mean ± SD (normal distribution, Student's *t*‐test) or median [interquartile range] (non‐normal, Mann–Whitney). Categorical variables: Pearson *χ* ^2^ or Fisher's exact test.

Table 2 shows the comparison of cardiometabolic indicators between the groups using the bilateral Mann–Whitney test. Participants with ST ≥ 8 h/day had significantly higher BMI values (29.26 ± 4.46 vs. 26.38 ± 4.09 kg/m^2^; *p* = 0.020), WHR (0.86 ± 0.10 vs. 0.80 ± 0.07; *p* = 0.011), SBP (138.33 ± 10.66 vs. 128.23 ± 9.59 mmHg; *p* = 0.003), DBP (89.29 ± 5.87 vs. 85.38 ± 4.07 mmHg; *p* = 0.016) and blood glucose (101.14 ± 5.67 vs. 96.67 ± 3.37 mg/dL; *p* = 0.001), in addition to significantly lower peripheral oxygen saturation (93.81 ± 3.59 vs. 96.18 ± 2.30%; *p* = 0.008). No significant difference was observed for resting heart rate.

**Table 2:** Comparison of cardiometabolic indicators according to daily sitting time (mean ± SD).

Variable	ST ≥ 8 h/day (*n* = 21) Mean ± SD	ST < 8 h/day (*n* = 39) Mean ± SD	Diff. average	*p* value
BMI (kg/m^2^)	29.26 ± 4.46	26.38 ± 4.09	2.88	0.020*
WHR	0.86 ± 0.10	0.80 ± 0.07	0.06	0.011*
SBP (mmHg)	138.33 ± 10.66	128.23 ± 9.59	10.10	0.003*
DBP (mmHg)	89.29 ± 5.87	85.38 ± 4.07	3.90	0.016*
HR (bpm)	79.95 ± 9.28	77.51 ± 6.52	2.44	0.166
Saturation (%)	93.81 ± 3.59	96.18 ± 2.30	–2.37	0.008*
Blood glucose (mg/dL)	101.14 ± 5.67	96.67 ± 3.37	4.48	0.001*

Abbreviations: BMI = body mass index, DBP = diastolic blood pressure, FC = heart rate (HR), HR = heart rate, PAD = diastolic blood pressure (DBP), RCQ = waist‐to‐hip ratio (WHR), SBP = systolic blood pressure, TS = sitting time, WHR = waist‐to‐hip ratio.

* *p* < 0.05 (bilateral Mann–Whitney test).

Table 3 shows the crude and partial Spearman correlations between ST and cardiometabolic indicators. In the crude analyses, positive and significant correlations were identified with WHR (*ρ* = +0.278; *p* = 0.031), SBP (*ρ* = +0.290; *p* = 0.024) and glycemia (*ρ* = +0.351; *p* = 0.006), and a significant negative correlation with peripheral saturation (*ρ* = –0.363; *p* = 0.004). After adjusting for the eight covariates by the residual method, the association with blood glucose was the only one that remained statistically significant (partial *ρ* = +0.279; *p* = 0.032). The adjusted models had acceptable VIFs (maximum VIF = 6.36; all < 10).

**Table 3:** Crude and partial Spearman's correlation between daily sitting time and cardiometabolic variables in adults in the Federal Court of Caraguatatuba—São Paulo.

Variable	Crude analysis			Adjusted analysis		
	*ρ*	IC95%	Crude *p*	*ρ* partial	Partial CI95%	Partial *p*
Age (years)	0.190	–0.054 to 0.410	0.146	—	—	—
BMI (kg/m^2^)	0.203	–0.054 to 0.434	0.120	–0.001	–0.253 to 0.251	0.993
WHR	0.278	0.026 to 0.497	0.031*	0.068	–0.180 to 0.308	0.607
SBP (mmHg)	0.290	0.039 to 0.507	0.024*	0.176	–0.071 to 0.401	0.179
DBP (mmHg)	0.228	–0.027 to 0.456	0.080	0.066	–0.181 to 0.306	0.618
HR (bpm)	0.075	–0.183 to 0.323	0.570	0.057	–0.190 to 0.297	0.666
Saturation (%)	–0.363	–0.565 to –0.121	0.004*	–0.163	–0.398 to 0.090	0.213
Blood glucose (mg/dL)	0.351	0.107 to 0.556	0.006*	0.279	0.025 to 0.499	0.032*

*Note:* 95%CI of crude correlations estimated by Fisher transformation z (consistent with Spearman's *p*: CI excludes zero when *p* < 0.05). 95%CI of partial correlations by the Fisher z transformation applied to the residues. The “adjusted analysis” column presents Spearman's partial correlations estimated by the residual method, adjusted for sex, age, physical activity, smoking, alcohol, medications, preexisting diseases, and stress. Gross *p* = unadjusted *p* value; crude *p* = unadjusted *p* value; partial *p* = after simultaneous adjustment. The variable age was not included in the partial analysis because it constitutes an adjustment covariate (“—”). Maximum VIF: 6.36; all < 10, although the maximum value observed suggests possible moderate collinearity in a small sample.

* Crude *p* < 0.05.

Table 4A presents the results of the unadjusted (simple) linear regression. ST was a significant predictor of peripheral saturation (*B *= –1.425; 95%CI: –2.483 to –0.368; *R* ^2^aj = 9.6%; *p* = 0.009) and blood glucose (*B* = +1.782; 95%CI: +0.066 to 3.498; *R* ^2^aj = 5.3%; *p* = 0.042), but not of the other indicators. Table 4B shows the adjusted multiple models.

**Table 4A:** Simple linear regression between daily sitting time and cardiometabolic variables in adults from the Federal Court of Caraguatatuba—São Paulo.

Dependent variable	B (coefficient)	IC95% (inf – sup)	*R* ^2^ aj (%)	*p* value
BMI (kg/m^2^)	0.462	–1.174 to 2.098	–1.2	0.574
WHR	0.022	–0.010 to 0.053	1.5	0.171
SBP (mmHg)	2.622	–1.422 to 6.667	1.1	0.199
DBP (mmHg)	1.020	–0.855 to 2.896	0.3	0.281
HR (bpm)	–0.759	–3.587 to 2.070	–1.2	0.593
Saturation (%)	–1.425	–2.483 to –0.368	9.6	0.009*
Blood glucose (mg/dL)	1.782	0.066 to 3.498	5.3	0.042*

Abbreviations: B = non‐standardized regression coefficient, BMI = body mass index, DBP = diastolic blood pressure, FC = heart rate (HR), HR = heart rate, PAD = diastolic blood pressure (DBP), *R* ^2^aj = adjusted coefficient of determination, RCQ = waist‐to‐hip ratio (WHR), SBP = systolic blood pressure, WHR = waist‐to‐hip ratio.

* *p* *<* 0.05.

**Table 4B:** Adjusted multiple linear regression: sitting time as an independent variable, controlled for sex, age, physical activity, smoking, alcohol, medications, preexisting diseases, and stress.

Dependent variable	*B* adjusted	IC95% (inf – sup)	*R* ^2^ aj (%)	*p* value
BMI (kg/m^2^)	0.215	(–1.718; 2.148)	15.7	0.824
WHR	0.010	(–0.025; 0.044)	29.4	0.570
SBP (mmHg)	1.853	(–2.249; 5.954)	39.3	0.369
DBP (mmHg)	0.408	(–1.719; 2.536)	23.4	0.702
HR (bpm)	0.215	(–3.240; 3.670)	9.8	0.901
Saturation (%)	–1.243	(–2.647; 0.161)	5.0	0.081
Blood glucose (mg/dL)	1.905	(–0.256; 4.066)	10.4	0.083

*Note:* No coefficient reached *p* < 0.05 after full adjustment. Blood glucose showed the largest adjusted coefficient (*B* = +1.905 mg/dL per hour of sitting time; *p* = 0.083; 95%CI: –0.256 to 4.066); the wide confidence interval precludes conclusions about clinical relevance.

Abbreviations: *B* = non‐standardized regression coefficient, R^2^ aj = adjusted coefficient of determination of the multiple model.

Table 5 presents the simple prevalence ratios with validated clinical cutoff points. The group with ST ≥ 8 h/day had a higher prevalence of WHR elevated according to the WHO sex‐specific criteria (PR = 6.19; 95%CI: 1.91–20.07; *p* = 0.001), peripheral saturation below 95% (PR = 2.79; 95%CI: 1.36–5.73; *p* = 0.010) and altered blood glucose (PR = 3.45; 95%CI: 1.63–7.30; *p* = 0.002). BMI, SBP, and DBP did not show statistically significant associations with the clinical cut‐off point adopted.

**Table 5:** Prevalence ratio of cardiometabolic alterations according to daily sitting time in adults in the Federal Court of Caraguatatuba—São Paulo.

Variable	ST ≥ 8 h/day (*n* = 21)				ST < 8 h/day (*n* = 39)				PR (95% CI)	*p* value
	Changed (%)	*n*	Normal (%)	*n*	Changed (%)	*n*	Normal (%)	*n*		
BMI (kg/m^2^)	81.0%	17	19.0%	4	64.1%	25	35.9%	14	1.26 (0.92–1.73)	0.241
WHR	47.6%	10	52.4%	11	7.7%	3	92.3%	36	6.19 (1.91–20.07)	0.001*
SBP (mmHg)	66.7%	14	33.3%	7	51.3%	20	48.7%	19	1.30 (0.85–2.00)	0.382
DBP (mmHg)	76.2%	16	23.8%	5	66.7%	26	33.3%	13	1.14 (0.82–1.58)	0.637
Saturation (%)	57.1%	12	42.9%	9	20.5%	8	79.5%	31	2.79 (1.36–5.73)	0.010*
Blood glucose (mg/dL)	61.9%	13	38.1%	8	17.9%	7	82.1%	32	3.45 (1.63–7.30)	0.002*

*Note:* PR = simple prevalence ratio, obtained by the ratio between the prevalences of the groups. 95%CI calculated by the log‐normal method for Table 2×2. The *p* value was obtained using Pearson's *χ* ^2^ test; for BMI and WHR, Fisher's exact test was used due to an expected frequency lower than 5 in any cell. Poisson regression with robust variance was not used due to the small number of events in some categories. Any discrepancies between the 95%CI and the *p* value result from the use of different methods (log‐normal CI and exact/*χ* ^2^ tests), a common practice in epidemiological Table 2×2 with a small number of events. For WHR, sex‐specific cut‐off points were adopted: ≥ 0.90 for men and ≥ 0.85 for women (high cardiovascular risk, WHO, 2008). For Peripheral Saturation, the “Above (%)” column refers to the proportion of participants with SpO2 below 95% (mild hypoxemia); higher proportion indicates worse outcome. Other cut‐off points: BMI ≥ 25 kg/m^2^ (overweight, WHO); SBP ≥ 130 mmHg (AHA/ACC 2017); DBP ≥ 85 mmHg (AHA/ACC 2017); Blood glucose ≥ 100 mg/dL (altered fasting glucose, ADA 2024). HR was excluded due to the absence of a clinically justifiable cutoff point.

* *p* *<* 0.05.

## 4 Discussion

The findings of the present study suggest an association between longer sitting time and a less favorable cardiometabolic profile in adult public servants. The group with sitting time equal to or greater than 8 h/day presented, in the unadjusted comparative analyses, a less favorable health profile in six of the seven outcomes evaluated, with significant differences in systolic blood pressure, blood glucose, waist‐to‐hip ratio, BMI, and peripheral oxygen saturation. The entire sample had sitting time of at least 6 h/day, indicating that these workers are chronically exposed to sedentary behavior at levels that meet or exceed the sitting time levels associated with increased risk according to international evidence [18, 19]. It is worth noting that, in Brazil, studies investigating the relationship between sitting time and cardiometabolic outcomes in populations of adult workers are still scarce. Most of the national literature on sedentary behavior is based on data from general population surveys [15, 16], without control for multiple clinical confounding variables and without focus on specific occupational groups such as public servants. In this sense, the present findings represent an original contribution that expands the knowledge about the cardiometabolic risks associated with sedentary behavior in the Brazilian context.

At the international level, a systematic review by Reichel et al. [21] found no clear association between occupational sedentary behavior and cardiovascular outcomes across many studies analyzed, underscoring the heterogeneity of evidence and methodological challenges. The physical activity paradox described by Holtermann et al. [22] also highlights that occupational physical activity may not confer the same cardiovascular benefits as leisure‐time activity, a concept relevant when interpreting findings in occupational settings.

Systolic blood pressure was the indicator with the most significant absolute difference between the groups, with 10.1 mmHg separating the servers with the longest and lowest sitting time (*p* = 0.003), a difference that has clinical relevance in magnitude. However, this association lost significance after adjusting for covariates, suggesting the influence of confounders such as gender, age, and behavioral factors. The AHA/ACC guidelines estimate that 5 mmHg reductions in SBP are associated with about a 10% reduction in cardiovascular risk [4, 5], which contextualizes the finding, although its statistical independence has not been confirmed. Beunza et al. [10], in a prospective cohort with 6742 university students followed up over 6 years, observed that individuals with more sitting time had a 48% higher risk of developing arterial hypertension, regardless of the level of physical activity practiced. In the present study, the association between sitting time and SBP did not remain significant after adjusting for the covariates, a result that can be partially explained by the use of medications reported by 31.7% of the participants, for various conditions, which may have interfered with the outcomes in a non‐systematic way, attenuating differences between the groups after adjustment. This type of pharmacological confounding is recognized as one of the main methodological challenges in observational studies on blood pressure in adult populations. The review by O'Brien et al. [11], which analyzed 40 systematic reviews on the subject, confirms that prospective studies identify the association more consistently than cross‐sectional studies, which is expected given the greater control of confounders and the possibility of establishing temporality. The RESET‐BP clinical trial [23] provides the most direct experimental support available: the reduction of sitting time in the work environment over 12 weeks produced a significant decrease in systolic blood pressure in office workers, which biologically validates the causality hypothesis that the present study cannot establish.

Blood glucose was the outcome with the most informative behavior in this study. In addition to the significant difference in the comparison between groups (101.14 vs. 96.67 mg/dL; *p* = 0.001), it was the only outcome that remained associated with sitting time in the adjusted partial correlation analyses (partial *ρ* = 0.279; *p* = 0.032). However, this association did not reach statistical significance in multiple regression (adjusted *B* = 1.905 mg/dL per hour of sitting time; *p* = 0.083; 95%CI: −0.256 to 4.066), which prevents full statistical independence from being affirmed. Physiological plausibility remains solid: prolonged sitting suppresses muscle LPL activity and inhibits GLUT‐4 translocation, reducing insulin‐independent glucose uptake [7]. Pinto et al. [8], in a review published in Physiological Reviews, demonstrated that the metabolic effects of sedentary behavior are not entirely reversed by the practice of physical exercise. Bergouignan et al. [9] confirmed that only 3 days of regular interruptions of sitting time were sufficient to modulate glucose uptake pathways, which biologically supports the association observed in the partial correlation.

The magnitude of the association with blood glucose is noted in the context that the sample had a mean blood glucose of 98.7 mg/dL, a value very close to the threshold of 100 mg/dL established for the diagnosis of impaired fasting glucose. In this context, within the narrow range of sitting time observed in this sample, participants with higher ST tended to have glycemic values closer to the threshold for altered blood glucose, which may have clinical relevance in sedentary occupational populations. Brakenridge et al. [24] confirmed, in a study with adults with and without type 2 diabetes, that both the total volume of sitting time and the duration of continuous sitting sessions are independently associated with worse glycemic outcomes, with the most significant benefits when sitting time is replaced by low‐intensity activity. Smith et al. [25], in a meta‐analysis of randomized clinical trials with adults with type 2 diabetes, demonstrated that interventions to reduce sitting time produced a clinically significant improvement in glycemic control over 8–12 weeks, without the participants necessarily increasing the volume of formal exercise. Mingyue et al. [20], in a systematic review with meta‐analysis of crossover trials, identified that the most effective strategy consists of interrupting sitting time every 30 min with light walking breaks of 1–5 min, which significantly reduced postprandial blood glucose and the area under the insulin curve. This evidence has a direct and low‐cost practical implication for the routine of public servants on administrative work, given that short and regular breaks are feasible without prejudice to productivity.

The loss of significance of the associations with SBP, WHR, and peripheral saturation in the adjusted analyses can be understood in the light of two complementary aspects. From the point of view of confounding, men simultaneously had more sitting time and higher WHR in this sample, so that when adjusting for sex, part of the effect attributable to sitting time is absorbed by this covariate. The use of medications, in turn, affects SBP so that adjusting for this variable reduces the detectable difference between groups. From a statistical point of view, the available sample size may have reduced the ability to detect adjusted associations of lower magnitude. For WHR specifically, part of the crude association may reflect sex differences in the adiposity distribution, given that men simultaneously had higher ST and higher WHR in this sample. That the effect on WHR exists in larger samples is supported by the meta‐analysis by Silveira et al. [26], with more than 638,000 participants, who recorded a significant association between sedentary behavior and abdominal obesity (OR = 1.45; 95%CI: 1.21–1.75). The dose‐response meta‐analysis by Wu et al. [14] demonstrated that prolonged sedentary behavior is associated with a 71% higher risk of metabolic syndrome in the categories with the highest exposure.

One finding that deserves reflection is that 17.1% of the participants classified as sufficiently active, with physical activity equal to or greater than 150 min/week, had sitting time equal to or greater than 8 h/day. This data illustrates what the literature has been describing as the “active couch potato” paradox [27]: complying with physical activity recommendations does not confer automatic protection against the adverse effects of prolonged time in a sitting posture. The meta‐analysis by Ekelund et al. [28], conducted with data from more than one million adults from four continents, demonstrated that around 60–75 min of moderate physical activity per day are necessary to completely attenuate the risk of mortality associated with high sitting time, a volume much higher than that practiced by the average of this sample. Blodgett et al. [29], using objective accelerometry in more than 15,000 participants of the ProPASS Consortium, reinforced that it is the composition of movement throughout the 24 h of the day, and not only the volume of planned exercise, that is the most relevant determinant of cardiovascular risk. These findings converge to a conclusion of direct interest to public health and occupational health managers: programs aimed at public servants need to integrate, in a complementary way, both the encouragement of regular physical exercise and strategies for frequent interruption of prolonged sitting throughout the day, such as scheduled movement breaks, standing periods, and behavioral cues to reduce continuous sitting. Although these interventions are often implemented in workplace settings, the present study measured total daily sitting time—not occupational sitting specifically—and therefore workplace recommendations should be considered contextually plausible rather than directly derived from the data.

The behavioral profile of this sample adds an additional element relevant to public health. The prevalence of self‐reported smoking (40.0%) was high in comparison with recent national estimates [15], although methodological differences between the surveys recommend caution in this comparison, in addition to the fact that alcohol consumption reported by 73.3% of the participants indicates an accumulation of behavioral risk factors that coexist with a sedentary lifestyle and that increase multicollinearity among the covariates of the adjusted models. The prevalence of medium or high stress of 68.3%, a variable rarely controlled in Brazilian studies on sedentary behavior, is another piece of data that deserves attention. Occupational stress can contribute to worse cardiometabolic outcomes both through direct neuroendocrine mechanisms, with activation of the hypothalamic‐pituitary‐adrenal axis and cortisol elevation, and indirectly, by favoring compensatory sedentary behaviors as an alternative to stress. Although stress was included as a covariate, its assessment by simple ordinal self‐report limits the accuracy of the measure compared to validated psychometric scales. The simultaneous inclusion of these variables in the fit model constitutes a methodological difference between this study and the national literature on sitting time.

A post‐hoc power analysis indicated that observed statistical power for the significant crude correlations ranged from 58% (WHR, *ρ *= 0.278) to 82% (SpO_2_, *ρ *= 0.363). The partial correlation with glucose (*ρ *= 0.279) had an estimated power of 58%, reinforcing the need for larger samples in adjusted models.

The study has limitations that should be considered in the interpretation of the findings. Although sleep duration and quality were collected, they were not included in the primary adjusted models, as they were not pre‐specified in the analytical plan; future studies should consider incorporating sleep as a covariate, given its established association with both sedentary behavior and cardiometabolic outcomes. The stress variable was assessed through a single ordinal self‐report item (low/medium/high), which may not capture occupational stress specifically and lacks the precision of validated psychometric instruments. The model with eight covariates may represent a risk of overfitting for a sample of 60 participants; a sensitivity analysis adjusting only for age and sex was not performed but is recommended in future studies to assess model stability. The cross‐sectional design prevents the establishment of causal relationships and is susceptible to reverse causality, given that individuals with poorer glycemic control or hypertension may modify their sitting time patterns in response to medical advice. The assessment of sitting time using the IPAQ short version, although validated for the Brazilian population [17], is a self‐report instrument subject to recall bias and unable to distinguish the continuous accumulation of sitting time from more fragmented patterns throughout the day, a distinction that has physiological relevance documented by studies with thigh accelerometry [24, 29]. The sample size of 60 participants, with eight adjustment covariates, may have been insufficient to detect associations of moderate magnitude after multiple control. Finally, the convenience sample of a single institution limits the generalization of the findings to other occupational contexts. Strengths include the direct and standardized evaluation of multiple cardiometabolic markers with calibrated equipment, the simultaneous inclusion of eight rarely controlled behavioral and clinical covariates together in the national literature, the estimation of the 95%CI correlations by the Fisher z transformation, and the original contribution on sedentary behavior in Brazilian public servants, a group for which there are practically no published data.

## 5 Conclusion

The present study suggests that more daily sitting time is associated with a less favorable cardiometabolic profile in adult civil servants, especially in the unadjusted comparative analyses, in which higher values of BMI, WHR, blood pressure, and blood glucose were observed, in addition to lower peripheral saturation. After adjusting for clinical and behavioral covariates, the associations were attenuated, and blood glucose presented the most consistent sign, with significant partial correlation (partial *ρ* = 0.279; *p* = 0.032), although without full statistical confirmation in multiple regression (*p* = 0.083). These findings indicate that sitting time may represent a relevant marker of cardiometabolic risk in occupational settings, but should be interpreted with caution due to the cross‐sectional design, small sample size, low variability of self‐reported sitting time, and the use of self‐reported measures.

From a public health perspective, the findings are especially relevant in a context in which Brazilian studies on sitting time and cardiometabolic outcomes in adult workers are still scarce. The fact that the entire sample had sitting time of at least 6 h/day, with 35% of the employees above the threshold of 8 h/day, indicates that high daily sitting time represents a prevalent and potentially underdiagnosed cardiometabolic risk indicator in this occupational group. The practical implications of the results point to the need for occupational health policies in public institutions to incorporate, in addition to the encouragement of physical exercise, strategies for regular interruption of prolonged sitting throughout the day. Since sitting time was assessed as total daily sitting—not exclusively occupational—workplace interventions represent a contextually relevant but not directly operationalized implication of these findings. Longitudinal studies with appropriately sized samples, the use of objective measurements of sitting time by thigh accelerometry, and stratification by medication use and smoking profile are necessary to confirm the independence of the observed associations and to define the most clinically effective intervention thresholds for this group.

## Author Contributions

**Leonardo Lima:** conceptualization, investigation, validation, formal analysis, supervision. **Carlos Eduardo Rosa da Silva:** conceptualization, investigation, methodology, validation, formal analysis, software, data curation, visualization, writing – review and editing. **Erinaldo Andrade:** investigation, conceptualization, visualization, project administration, software. **Luis Carlos de Oliveira:** software. **Willian Santana:** methodology. **Jose Eduardo Hydes:** visualization. **Yuri Xavier:** investigation, writing – original draft, software, data curation. **Aylton Figueira:** conceptualization, investigation, funding acquisition, writing – original draft, methodology, validation, visualization, writing – review and editing, formal analysis, software, project administration, resources, supervision.

## Funding

The authors have nothing to report.

## Ethics Statement

This study was conducted in accordance with the Declaration of Helsinki and approved by the Research Ethics Committee of Universidade São Judas Tadeu (CEP/USJT, protocol no. 6.117.542). All participants provided written informed consent prior to data collection.

## Conflicts of Interest

The authors declare no conflicts of interest.

## Transparency Statement

The corresponding author (Carlos Eduardo Rosa da Silva) affirms that this manuscript is an honest, accurate, and transparent account of the study being reported; that no important aspects of the study have been omitted; and that any discrepancies from the study as planned have been explained.

## Data Availability

The data that support the findings of this study are available from the corresponding author upon reasonable request.

## References

[1] M. S. Tremblay , S. Aubert , J. D. Barnes , et al., “Sedentary Behavior Research Network (SBRN)— Terminology Consensus Project Process and Outcome,” International Journal of Behavioral Nutrition and Physical Activity 14, no. 1 (2017): 75. 28599680 10.1186/s12966-017-0525-8 PMC5466781

[2] A. Biswas , P. I. Oh , G. E. Faulkner , et al., “Sedentary Time and Its Association With Risk for Disease Incidence, Mortality, and Hospitalization in Adults: A Systematic Review and Meta‐Analysis,” Annals of Internal Medicine 162, no. 2 (2015): 123–132. 25599350 10.7326/M14-1651

[3] E. G. Wilmot , C. L. Edwardson , F. A. Achana , et al., “Sedentary Time in Adults and the Association With Diabetes, Cardiovascular Disease and Death: Systematic Review and Meta‐Analysis,” Diabetologia 55, no. 11 (2012): 2895–2905. 22890825 10.1007/s00125-012-2677-z

[4] L. F. M. Rezende , T. H. Sá , G. I. Mielke , J. Y. K. Viscondi , J. P. Rey‐López , and L. M. T. Garcia , “All‐Cause Mortality Attributable to Sitting Time,” American Journal of Preventive Medicine 51, no. 2 (2016): 253–263. 27017420 10.1016/j.amepre.2016.01.022

[5] G. A. Roth , G. A. Mensah , and V. Fuster , “The Global Burden of Cardiovascular Diseases and Risks,” Journal of the American College of Cardiology 76, no. 25 (2020): 2980–2981. 33309174 10.1016/j.jacc.2020.11.021

[6] A. B. Oliveira , P. T. Katzmarzyk , W. S. Dantas , I. J. M. Benseñor , A. C. Goulart , and U. Ekelund , “Profile of Leisure‐Time Physical Activity and Sedentary Behavior in Adults in Brazil: A Nationwide Survey, 2019,” Epidemiologia e Serviços de Saúde 32, no. 2 (2023): e2023168. 37585879 10.1590/S2237-96222023000200016 PMC10421589

[7] M. T. Hamilton , D. G. Hamilton , and T. W. Zderic , “Role of Low Energy Expenditure and Sitting in Obesity, Metabolic Syndrome, Type 2 Diabetes, and Cardiovascular Disease,” Diabetes 56, no. 11 (2007): 2655–2667. 17827399 10.2337/db07-0882

[8] A. J. Pinto , A. Bergouignan , P. C. Dempsey , et al., “Physiology of Sedentary Behavior,” Physiological Reviews 103, no. 4 (2023): 2561–2622. 37326297 10.1152/physrev.00022.2022 PMC10625842

[9] A. Bergouignan , C. Latouche , S. Heywood , et al., “Frequent Interruptions of Sedentary Time Modulates Contraction‐ and Insulin‐Stimulated Glucose Uptake Pathways in Muscle: Ancillary Analysis From Randomized Clinical Trials,” Scientific Reports 6 (2016): 32044. 27554943 10.1038/srep32044 PMC4995429

[10] J. J. Beunza , M. A. Martínez‐González , S. Ebrahim , et al., “Sedentary Behaviors and the Risk of Incident Hypertension: The SUN Cohort,” American Journal of Hypertension 20, no. 11 (2007): 1156–1162. 17954361 10.1016/j.amjhyper.2007.06.007

[11] M. W. O'Brien , M. E. Shivgulam , A. H. Domínguez , et al., “Impact of Sedentary Behaviors on Blood Pressure and Cardiovascular Disease: An Umbrella Review of Systematic Reviews and Meta‐Analyses,” Sports Medicine 54, no. 12 (2024): 3097–3110. 39162946 10.1007/s40279-024-02099-w

[12] F. R. Cavallo , C. Golden , J. Pearson‐Stuttard , C. Falconer , and C. Toumazou , “The Association Between Sedentary Behaviour, Physical Activity and Type 2 Diabetes Markers: A Systematic Review of Mixed Analytic Approaches,” PLoS One 17, no. 5 (2022): e0268289. 35544519 10.1371/journal.pone.0268289 PMC9094551

[13] T. J. Saunders , T. McIsaac , K. Douillette , et al., “Sedentary Behaviour and Health in Adults: An Overview of Systematic Reviews,” Applied Physiology, Nutrition, and Metabolism 45, no. 10S2 (2020): S197–S217. 10.1139/apnm-2020-0272 33054341

[14] J. Wu , H. Zhang , L. Yang , et al., “Sedentary Time and the Risk of Metabolic Syndrome: A Systematic Review and Dose‐Response Meta‐Analysis,” Obesity Reviews 23, no. 12 (2022): e13510. 36261077 10.1111/obr.13510

[15] G. I. Mielke , P. C. Hallal , G. B. A. Rodrigues , C. L. Szwarcwald , F. V. Santos , and D. C. Malta , “Prática De Atividade Física E Hábito De Assistir À Televisão Entre Adultos No Brasil: Pesquisa Nacional De Saúde 2013,” Epidemiologia e Serviços de Saúde 24, no. 2 (2015): 277–286.

[16] G. I. Mielke , I. C. M. da Silva , N. Owen , and P. C. Hallal , “Brazilian Adults' Sedentary Behaviors by Life Domain: Population‐Based Study,” PLoS One 9, no. 3 (2014): e91614. 24619086 10.1371/journal.pone.0091614 PMC3950247

[17] S. Matsudo , T. Araújo , V. Matsudo , et al., “International Physical Activity Questionnaire (IPAQ): A Study of Validity and Reproducibility in Brazil,” Revista Brasileira de Atividade Física & Saúde 6, no. 2 (2001): 5–18.

[18] F. C. Bull , S. S. Al‐Ansari , S. Biddle , et al., “World Health Organization 2020 Guidelines on Physical Activity and Sedentary Behaviour,” British Journal of Sports Medicine 54, no. 24 (2020): 1451–1462. 33239350 10.1136/bjsports-2020-102955 PMC7719906

[19] P. T. Katzmarzyk , K. E. Powell , J. M. Jakicic , R. P. Troiano , K. Piercy , and B. Tennant , “Sedentary Behavior and Health: Update From the 2018 Physical Activity Guidelines Advisory Committee,” Medicine & Science in Sports & Exercise 51, no. 6 (2019): 1227–1241. 31095080 10.1249/MSS.0000000000001935 PMC6527341

[20] Y. Mingyue , L. Chen , W. Lili , et al., “Optimal Frequency of Interrupting Prolonged Sitting for Cardiometabolic Health: A Systematic Review and Meta‐Analysis of Randomized Crossover Trials,” Scandinavian Journal of Medicine & Science in Sports 34 (2024): e14769. 39630056 10.1111/sms.14769

[21] K. Reichel , M. Prigge , U. Latza , T. Kurth , and E. M. Backé , “Association of Occupational Sitting With Cardiovascular Outcomes and Cardiometabolic Risk Factors: A Systematic Review With a Sex‐Sensitive/Gender‐Sensitive Perspective,” BMJ Open 12, no. 2 (2022): e048017, 10.1136/bmjopen-2020-048017. PMC8830241 35135760

[22] A. Holtermann , N. Krause , A. J. van der Beek , and L. Straker , “The Physical Activity Paradox: Six Reasons Why Occupational Physical Activity (OPA) Does Not Confer the Cardiovascular Health Benefits That Leisure Time Physical Activity Does,” British Journal of Sports Medicine 52, no. 3 (2018): 149–150. 28798040 10.1136/bjsports-2017-097965

[23] B. Barone Gibbs , S. Perera , K. A. Huber , et al., “Effects of Sedentary Behavior Reduction on Blood Pressure in Desk Workers: Results From the RESET‐BP Randomized Clinical Trial,” Circulation 150, no. 5 (2024): 1416–1427. 39166323 10.1161/CIRCULATIONAHA.123.068564 PMC11512617

[24] C. J. Brakenridge , G. N. Healy , N. T. Hadgraft , et al., “Associations Between Sedentary Time, Sitting Patterns and Cardiometabolic Health in Adults With and Without Type 2 Diabetes,” Journal of Physical Activity & Health 21, no. 3 (2024): 153–163.

[25] S. Smith , B. Salmani , J. LeSarge , K. Dillon‐Rossiter , A. Morava , and H. Prapavessis , “Interventions to Reduce Sedentary Behaviour in Adults With Type 2 Diabetes: A Systematic Review and Meta‐Analysis,” PLoS One 19, no. 7 (2024): e0306439. 39078846 10.1371/journal.pone.0306439 PMC11288443

[26] E. A. Silveira , C. R. Mendonça , F. M. Delpino , et al., “Sedentary Behavior, Physical Inactivity, Abdominal Obesity and Obesity in Adults and Older Adults: A Systematic Review and Meta‐Analysis,” Clinical Nutrition ESPEN 50 (2022): 63–73. 35871953 10.1016/j.clnesp.2022.06.001

[27] N. Owen , G. N. Healy , C. E. Matthews , and D. W. Dunstan , “Too Much Sitting: The Population Health Science of Sedentary Behavior,” Exercise and Sport Sciences Reviews 38, no. 3 (2010): 105–113. 20577058 10.1097/JES.0b013e3181e373a2 PMC3404815

[28] U. Ekelund , J. Steene‐Johannessen , W. J. Brown , et al., Lancet Physical Activity Series 2 Executive Committee, Lancet Sedentary Behaviour Working Group , “Does Physical Activity Attenuate, or Even Eliminate, the Detrimental Association of Sitting Time With Mortality? A Harmonised Meta‐Analysis of Data From More Than 1 Million Men and Women,” Lancet 388, no. 10051 (2016): 1302–1310. 27475271 10.1016/S0140-6736(16)30370-1

[29] J. M. Blodgett , M. N. Ahmadi , A. J. Atkin , et al., “Device‐Measured 24‐Hour Movement Behaviors and Blood Pressure: A 6‐Part Compositional Individual Participant Data Analysis in the ProPASS Consortium,” Circulation 5 (2024): 2, 10.1161/circulationaha.124.069820. PMC11732261 39504653

